# Mild Electrical Stimulation with Heat Shock Reduces Visceral Adiposity and Improves Metabolic Abnormalities in Subjects with Metabolic Syndrome or Type 2 Diabetes: Randomized Crossover Trials

**DOI:** 10.1016/j.ebiom.2014.11.001

**Published:** 2014-11-11

**Authors:** Tatsuya Kondo, Kaoru Ono, Sayaka Kitano, Rina Matsuyama, Rieko Goto, Mary Ann Suico, Shuji Kawasaki, Motoyuki Igata, Junji Kawashima, Hiroyuki Motoshima, Takeshi Matsumura, Hirofumi Kai, Eiichi Araki

**Affiliations:** a Department of Metabolic Medicine, Faculty of Life Sciences, Kumamoto University, Kumamoto, Japan; b Department of Molecular Medicine, Faculty of Life Sciences, Kumamoto University, Kumamoto, Japan

**Keywords:** Metabolic syndrome, Type 2 diabetes, Heat shock response, Insulin resistance, Chronic inflammation

## Abstract

**Background:**

The induction of heat shock protein (HSP) 72 by mild electrical stimulation with heat shock (MES + HS), which improves visceral adiposity and insulin resistance in mice, may be beneficial in treating metabolic syndrome (MS) or type 2 diabetes mellitus (T2DM).

**Methods:**

Using open-label crossover trials, 40 subjects with MS or T2DM were randomly assigned using computer-generated random numbers to 12 weeks of therapeutic MES + HS followed by 12 weeks of no treatment, or vice versa. During the intervention period, physical and biochemical markers were measured.

**Findings:**

Compared to no treatment, MES + HS treatment was associated with a significant decrease in visceral adiposity (− 7.54 cm^2^ (− 8.61%), 95% CI − 8.55 to − 6.53 (p = 0.037) in MS, − 19.73 cm^2^ (− 10.89%), 95% CI − 20.97 to − 18.49 (p = 0.003) in T2DM). Fasting plasma glucose levels were decreased by 3.74 mg/dL (− 5.28%: 95% CI − 4.37 to − 3.09 mg/dL, p = 0.029) in MS and by 14.97 mg/dL (10.40%: 95% CI − 15.79 to 14.15 mg/dL, p < 0.001) in T2DM, and insulin levels were also reduced by 10.39% and 25.93%, respectively. HbA1c levels showed a trend toward reduction (− 0.06%) in MS, and was significantly declined by − 0.43% (95% CI − 0.55 to − 0.31%, p = 0.009) in T2DM. HbA1c level of less than 7.0% was achieved in 52.5% of the MES + HS-treated T2DM patients in contrast to 15% of the non-treated period. Several insulin resistance indices, inflammatory cytokines or adipokines, including C-reactive protein, adiponectin, and tumor necrosis factor-α, were all improved in both groups. In isolated monocytes, HSP72 expression was increased and cytokine expression was reduced following MES + HS treatment. Glucose excursions on meal tolerance test were lower after using MES + HS in T2DM.

**Interpretation:**

This combination therapy has beneficial impacts on body composition, metabolic abnormalities, and inflammation in subjects with MS or T2DM. Activation of the heat shock response by MES + HS may provide a novel approach for the treatment of lifestyle-related diseases.

**Funding:**

Funding for this research was provided by MEXT KAKENHI (Grants-in-Aid for Scientific Research from Ministry of Education, Culture, Sports, Science and Technology, Japan).

## Highlights

• We report the use of mild electrical stimulation with heat shock (MES + HS) in treating metabolic syndrome or type 2 diabetes.
• The treatment improves visceral adiposity, glucose homeostasis, insulin resistance and chronic inflammatory status.
• Our findings suggest that MES + HS might be a valuable therapeutic option for patients with these lifestyle-related diseases.

## 1 Introduction

Although DCCT, UKPDS and our Kumamoto Study (Shichiri et al., 2000) have shown that strict glycemic control could prevent microvascular complications, the increase of diabetes is still an important issue worldwide. The increase of type 2 diabetes mellitus (T2DM) is associated with excess visceral adiposity, which is tightly linked to metabolic syndrome (MS). MS is recognized as a cluster of cardiovascular risk factors such as hyperglycemia, dyslipidemia, elevated blood pressure and chronic inflammation (Alberti et al., 2005). Visceral fat has been demonstrated to express more pro-inflammatory cytokines than subcutaneous fat in obese states (Ohman et al., 2009). Inflammatory markers such as C-reactive protein (CRP) (Tamakoshi et al., 2003) and tumor necrosis factor (TNF)-α have been linked to MS.

Currently, there are no medications or modalities to reduce both visceral adiposity as well as chronic inflammation in MS or T2DM subjects. Recently, we (Morino et al., 2008a, Adachi et al., 2010) and others (Chung et al., 2008, Gupte et al., 2009) have shown that the beneficial metabolic advantages of heat shock response (HSR) activation, which appears to mainly involve heat shock protein (HSP) 72. HSP72 induction by mild electrical stimulation (MES) with heat shock (HS) (Morino et al., 2008a, Kondo et al., 2012), a transgenic system (Chung et al., 2008), heat treatment (Gupte et al., 2009, Kavanagh et al., 2011) or chemical inducers (Adachi et al., 2010) ameliorated abnormal metabolic features in animal models of T2DM, such as insulin resistance, hyperglycemia and visceral fat accumulation. Mild electrical stimulation enhances heat induction of HSP72 (Morino et al., 2008b) and may directly activate insulin signaling by modulating the insulin receptor localization of membrane components (Yano et al., 2010, Morino-Koga et al., 2013). In this study, we investigated the effects of MES + HS on glucose homeostasis, insulin resistance, visceral adiposity and inflammatory cytokine levels in male subjects with MS or T2DM. In addition, inflammatory characteristics of circulating monocytes were examined. This novel combination therapy may provide an additional treatment strategy to improve metabolic abnormalities in lifestyle-related diseases.

## 2 Materials and Methods

### 2.1 Study Participants

A total of 40 Japanese males with MS or T2DM were recruited. MS was defined by the Examination Committee for Criteria of Metabolic Syndrome and was diagnosed on the basis of the criteria of the American Diabetes Association. The study protocol conformed to the ethical guidelines of the Declaration of Helsinki, and written informed consent was obtained from each subject. These researches were approved by the Ethics Review Committee at Kumamoto University (Advanced Ethics No. 736 and Ethics No. 514). These clinical trials were registered with an approved ICMJE clinical trial registry, UMIN (ID: UMIN 000001149, 000003210 and 000007792). Protocol details can be found in the Supplemental materials.

### 2.2 MES + HS Treatment

The devices (BioMetronome®) for producing MES + HS were provided by Tsuchiya Rubber Co. Ltd. (Kumamoto, Japan). The description of the MES + HS device has been provided previously (Kondo et al., 2010). Briefly, MES + HS produces electrical stimulation of 1.4 ± 0.1 V/cm: the pads were positioned on the front and back of the abdomen, 55 pulses per second, 0.1 millisecond duration with 42 °C heat. The padded area was 15 cm in length × 25 cm in width.

### 2.3 Randomization and Masking

Forty eligible MS or T2DM subjects were randomly assigned using computer-generated random numbers into two groups by Latin square method, each containing 20 subjects. Neither subjects nor investigators were masked to treatment allocation at the time of enrollment.

### 2.4 Study Design and Clinical Protocol

This study was a prospective, randomized, open-label, crossover trial. Forty eligible MS or T2DM subjects were randomly assigned into two groups, each containing 20 subjects. Group I underwent a 12-week intervention period of MES + HS followed by 12 weeks with no treatment. The order was reversed in group II. During the MES + HS-treatment period, subjects were instructed to use MES + HS 4 times a week for 60 min per session. Exercise and diet alterations were prohibited during the entire period. At 0, 12 and 24 weeks, body compositions, abdominal adiposity, metabolic and biochemical examination with a 75 g oral glucose tolerance test (OGTT) in MS or a 592 kcal meal tolerance test (MTT) in T2DM were performed. The primary endpoint is the amount of visceral adiposity and glucose control. Other outcomes include blood pressure, insulin resistance, inflammatory cytokine levels and the HbA1c achievement ratio of less than 7.0%. For the primary outcome, we estimated the need to enroll 36 subjects to detect changes in visceral fat area of 15% with MES + HS as compared to no treatment, with statistical power of 80%, allowing for a type I (α) error of 0.05. Allowing for a loss to follow-up rate of 10%, 40 subjects were required to undergo randomization.

### 2.5 Quantification of Adiposity

Visceral fat area (VFA) and subcutaneous fat area (SFA) at the umbilical level were measured using CT scans (HiSpeed NX/i, GE Healthcare Japan Co., Ltd., Tokyo, Japan).

### 2.6 Insulin Sensitivity Indices

Several indices were calculated based on the results of OGTT in MS. The quantitative insulin-sensitivity check index (QUICKI), homeostasis model assessment of insulin resistance (HOMA-IR), HOMA-β, composite whole body insulin sensitivity index (cWBISI) and insulinogenic index (I.I.) were determined, as described previously (Yamashiro et al., 2010).

### 2.7 Monocytes Isolation and Analysis

To investigate the characteristics of monocyte in MS or T2DM subjects, 10 subjects were randomly selected. Before and after 4 weeks of MES + HS treatment, blood samples were collected during a fasted state. First, peripheral blood mononuclear cells (PBMCs) were isolated using BD Vacutainer™ CPT™ (BD, Franklin Lakes, NJ). Monocytes were subsequently isolated from the PBMCs magnetically by depletion technique (Miltenyl Biotech. Auburn. CA). For some experiments, monocytes were examined before and after activation with lipopolysaccharide (LPS: 160 ng/mL) overnight.

### 2.8 mRNA Expression Determined by qRT-PCR

Real time qRT-PCR was performed using mRNA from isolated monocytes stimulated with LPS. The protocol of qRT-PCR has been described previously (Adachi et al., 2010).

### 2.9 Statistical Analysis

Statistical analysis was performed with SPSS software (IBM, Chicago, IL, USA). All values were expressed as mean ± standard deviation (S.D.). The treatment effects of MES + HS were analyzed by paired a *t*-test if data were normally distributed or a Wilcoxon signed-rank test if not. Sequential changes were analyzed by repeated-measures ANOVA. Two-sided p-values of less than 0.05 were considered to indicate statistical significance.

## 3 Results

### 3.1 Characteristics of MS or T2DM Subjects

**Fig. 1:**
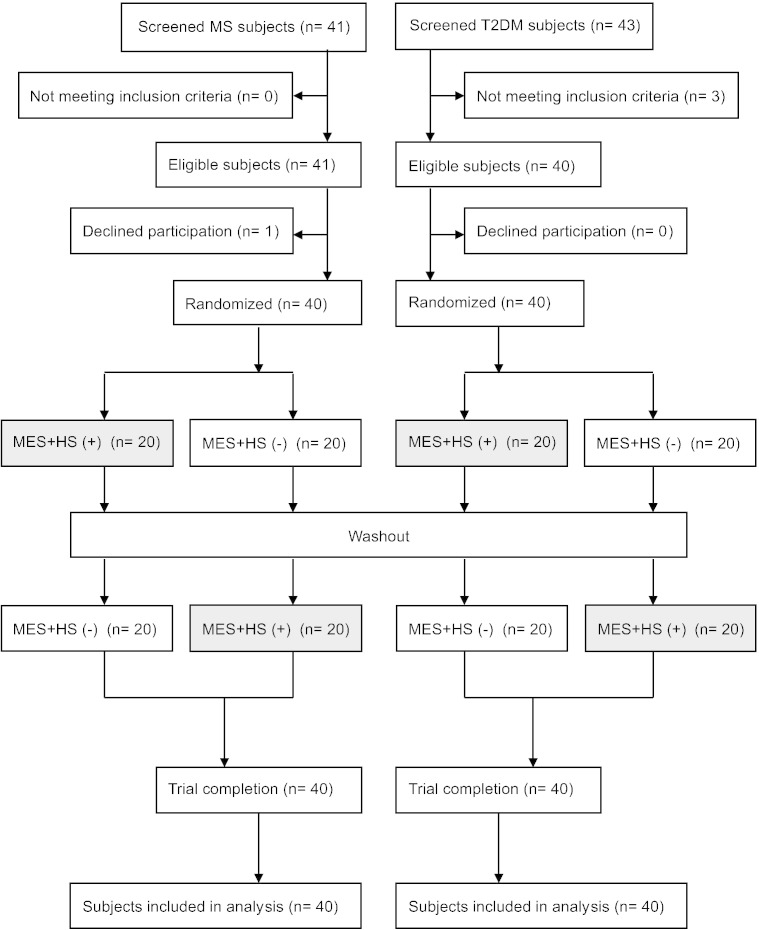
Flowchart of study subjects.

Japanese males diagnosed with MS or T2DM were randomly assigned to either group I or group II. Thereafter, subjects were randomized to one of two possible group sequences to receive no treatment or MES + HS treatment (Fig. 1). The baseline values were similar between the two groups (*n* = 40) (Table 1).

### 3.2 Adverse Effects

There were no harmful or adverse events including hypoglycemia or biochemical abnormalities during these studies. As only mild electrical current was used, no perceptible muscle contraction or muscle pain was generated in the subjects.

### 3.3 Adipose Tissue Composition and Physical Status

Visceral and subcutaneous adiposity were analyzed using abdominal CT scans. After MES + HS treatment, visceral fat area (VFA) in MS and T2DM was significantly decreased by 12.69 ± 3.25 cm^2^ (− 8.61%, p = 0.037, Table 2) and 20.88 ± 4.00 cm^2^ (− 10.89%, p = 0.003. Table 2), respectively. Although there was no change in subcutaneous fat area (SFA) as a result of MES + HS treatment, total abdominal adiposity was significantly reduced (Table 2).

Waist circumference (Wc) also showed similar changes associated with MES + HS treatment (Table 2). Although there was no significant change in MS, body mass index (BMI) was significantly decreased by 1.42% (p = 0.027) in T2DM. Systolic or diastolic blood pressure (SBP or DBP) was significantly reduced both in MS and T2DM (Table 3).

### 3.4 Glucose Homeostasis and Insulin Resistance

**Fig. 2:**
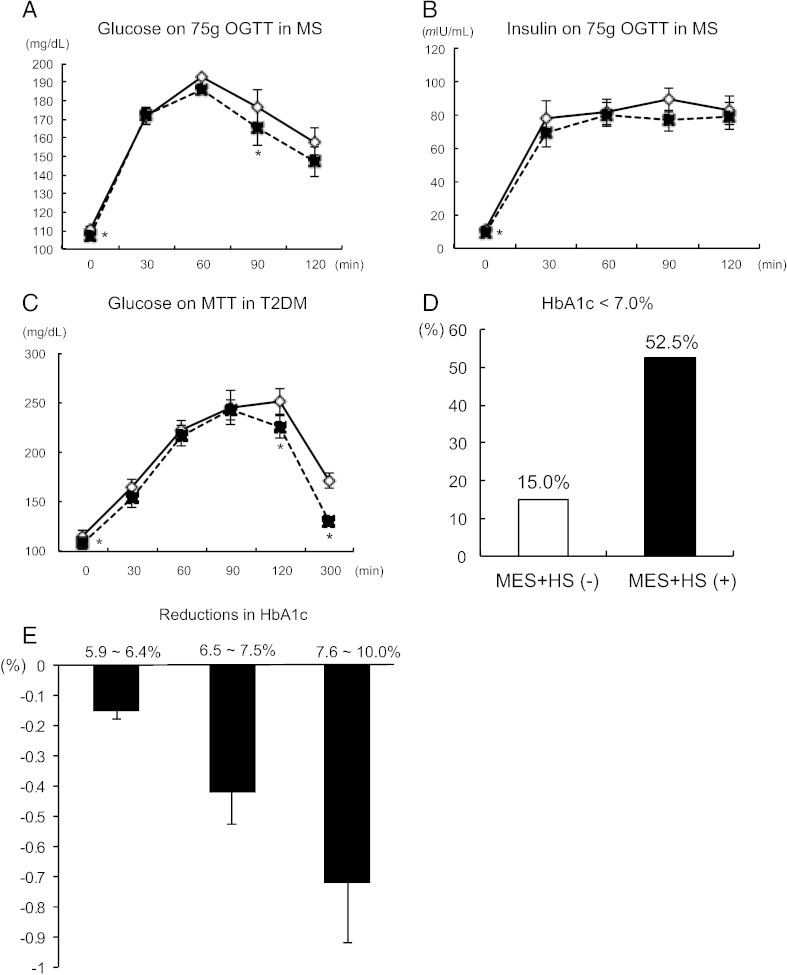
Glucose homeostasis in MES + HS-treated MS or T2DM subjects. (A) Glucose excursions on 75 g oral glucose tolerance test (OGTT) in MS subjects (n = 40) after no treatment (open diamonds with solid line) or MES + HS treatment (closed squares with dotted line) period. (B) Serum insulin response on 75 g OGTT in MS after no treatment or MES + HS treatment period. (C) Glucose excursions on 592 kcal of meal tolerance test (MTT) in T2DM patients (n = 40) after no treatment or MES + HS treatment period. (D) The ratio of HbA1c less than 7.0% in T2DM patients without MES + HS (MES + HS(−)) or with MES + HS treatment period (MES + HS(+)). (E) Reductions in HbA1c sorted by pre-HbA1c levels upon MES + HS treatment. Sequential changes were analyzed by repeated-measures ANOVA, and the differences at each time point were analyzed by unpaired *t*-test. *p < 0.05, **p < 0.01.

Fasting plasma glucose (FPG) was significantly decreased (− 5.95 mg/dL in MS, − 15.45 mg/dL in T2DM, Table 2). Fasting immuno-reactive insulin (IRI) showed similar alterations (− 1.08 μIU/mL in MS, − 3.89 μIU/mL in T2DM, Table 2). As these FPG and IRI were both reduced, HOMA-IR was significantly decreased (Table 2). QUICKI showed a trend of reduction by 2.70% (p = 0.09), and cWBISI was significantly increased (+ 20.61%, p = 0.029) in MS subjects. Upon 75 g-OGTT, FPG and glucose levels at 90 min were significantly lower than those at the non-treatment period (Fig. 2A). The insulin secreting indices, I.I. and HOMA-β were also evaluated. Although IRI response against glucose load was quite similar to the non-treatment period, I.I. showed a significant decrease in MS subjects (Table 2 and Fig. 2B). HOMA-β was unchanged during the study.

HbA1c (NGSP) levels showed a trend toward reduction (− 0.07 ± 0.04%, p = 0.143) in MS, and this was significantly decreased (− 0.49 ± 0.06%) from baseline in T2DM. Compared with no treatment period, HbA1c showed a significant reduction by 0.43% (p = 0.009). The glucose levels at 0, 120 and 300 min on meal tolerance test were significantly lower (0 min; 115.5 vs 108.7 mg/dL, p = 0.039. 120 min; 251.2 vs 225.4 mg/dL, p = 0.048. 300 min; 171.8 vs 130.1 mg/dL, p = 0.041. Fig. 2C) in T2DM. The proportion of patients who achieved the HbA1c goal of less than 7.0% was 52.5% in the MES + HS-treated period in contrast to 15.0% in the untreated period (Fig. 2D). Higher pre-HbA1c indicated greater reductions upon MES + HS treatment (HbA1c 5.9–6.4%: − 0.15%, 6.5–7.5%: − 0.42%, 7.6–10.0%: − 0.72%. Fig. 2E)

### 3.5 Inflammatory Cytokines and Adipokines

Visceral adiposity influences the production of inflammatory cytokines and adipokines. High sensitivity CRP significantly decreased by 53.85% in MS (p = 0.025) and by 4.54% in T2DM (p = 0.047) following MES + HS treatment. Adiponectin increased by 8.36% and 6.63% in MS and T2DM, respectively. Leptin tended to decrease by 8.89% in MS, and displayed a significant reduction by 10.61% in T2DM. TNF-α dropped by 8.97% and 21.60% in MS and T2DM, respectively. Interleukin-6 showed a significant reduction in MS (− 28.33%), but not in T2DM (− 2.50%).

### 3.6 Other Metabolic Benefits

To evaluate liver fat accumulation non-invasively, the AST/ALT ratio and liver/spleen attenuation (L/S) ratio were calculated. In parallel with the reduction of visceral adiposity by MES + HS treatment, the AST/ALT ratio increased with the treatment (Table 3). The L/S ratio showed a similar alteration (Table 3).

LDL-cholesterol was decreased as shown in Table 3. HDL-C was increased only in T2DM subjects (Table 3). Although BUN was not changed, serum creatinine (Cre) was slightly but significantly decreased in T2DM. Urinary excretion of albumin adjusted by urinary Cre was also reduced in T2DM.

### 3.7 Inflammatory Characteristics of Monocytes

**Fig. 3:**
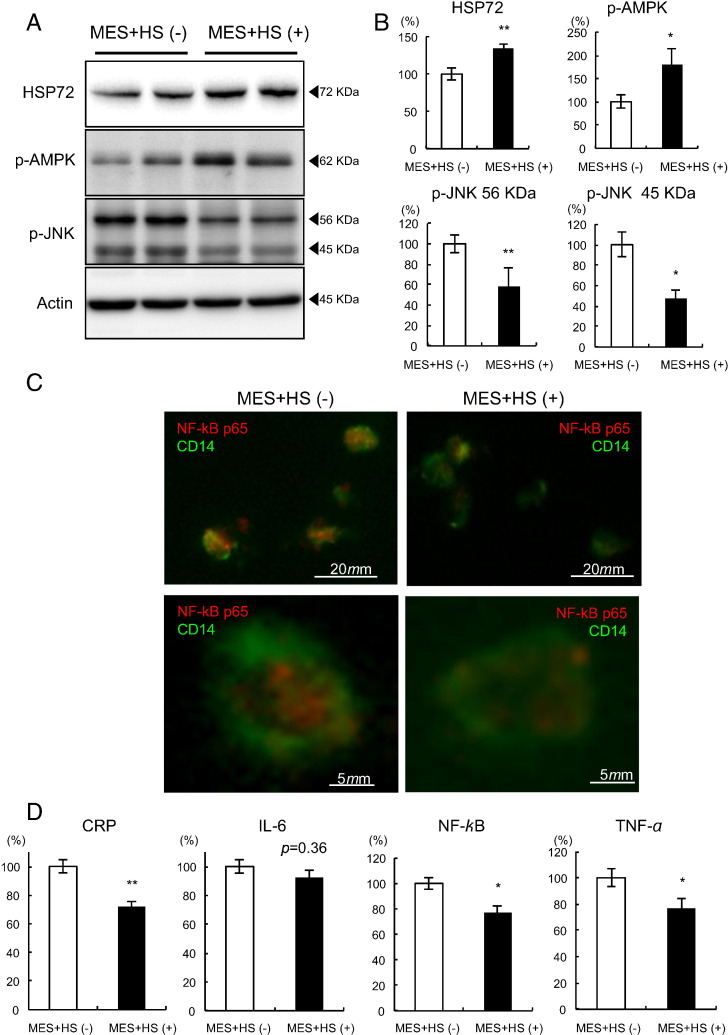
Inflammatory characteristics in CD14 positive monocytes in MS subjects. Ten MS subjects were selected, and 4 weeks of MES + HS treatment was performed. Before and after the MES + HS treatment period, blood sampling was performed on a fasted state. Circulating monocytes were isolated, then HSP72 protein, p-AMPK, p-JNK and actin were determined by Western blot analysis. Representative results are shown in A. The relative intensity calculated using actin is shown in B. (C) Isolated monocytes were subjected to immunofluorescent staining after LPS stimulation using antibodies against NF-κB p65 (red) and CD14 (green). Representative results are shown under low (upper panels) or high (lower panels) magnifications. (D) Isolated monocytes after LPS stimulation were subjected to mRNA measurements of CRP, IL-6, NF-κB and TNF-α. Averages of each mRNA level with or without MES + HS treatment are shown. *p < 0.05, **p < 0.01.

**Fig. 4:**
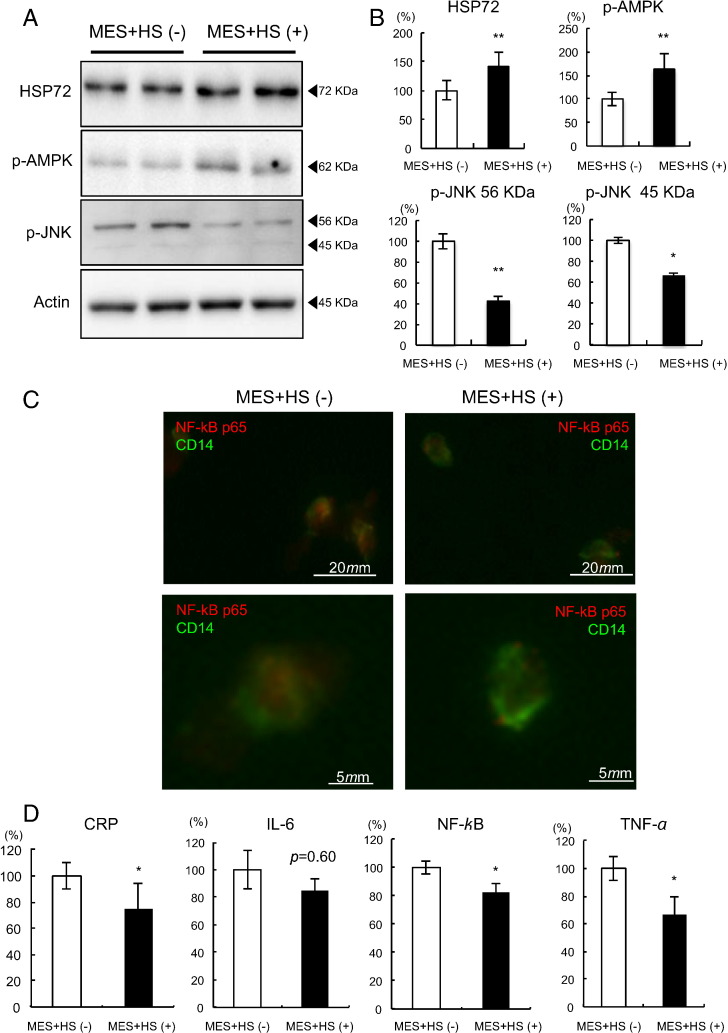
Inflammatory characteristics in CD14 positive monocytes in T2DM patients. Ten T2DM patients were selected, and 4 weeks of MES + HS treatment was performed. Before and after the MES + HS treatment period, blood sampling was performed on fasted state. Circulating monocytes were isolated, then HSP72 protein, p-AMPK, p-JNK and actin were determined by Western blot analysis. Representative results are shown in A. The relative intensity calculated using actin is shown in B. (C) Isolated monocytes were subjected to immunofluorescent staining after LPS stimulation using antibodies against NF-κB p65 (red) and CD14 (green). Representative results are shown under low (upper panels) or high (lower panels) magnifications. (D) Isolated monocytes after LPS stimulation were subjected to mRNA measurements of CRP, IL-6, NF-κB and TNF-α. Averages of each mRNA level with or without MES + HS treatment are shown. *p < 0.05, **p < 0.01.

To identify the effects of MES + HS on the inflammatory milieu in vivo, CD14 positive circulating monocytes were isolated, and levels of cytokine expression were examined both in MS and T2DM. After 4 weeks of MES + HS treatment, HSP72 protein levels in monocytes were significantly increased by 34.5% compared to baseline in MS (Fig. 3A and B. p < 0.001), and by 41.7% in T2DM (Fig. 4A and B. p < 0.001). Phosphorylation of AMP-kinase (AMPK) was also increased by 79.9% in MS (Fig. 3A and B. p = 0.0002), and by 63.2% in T2DM (Fig. 4A and B. p = 0.017). Phospho-c-jun N-terminal kinase (p-JNK) was attenuated by 42.1% in 56 KDa (p < 0.001) and by 52.6% in 45 KDa (p < 0.001) subunits in MS (Fig. 3A and B), and by 57.8% (p = 0.0002) and by 32.3% (p = 0.048), respectively in T2DM (Fig. 4A and B). LPS-stimulated NF-κB p65 subunit nuclear translocation, visualized by immunofluorescence, was attenuated in both MS and T2DM subjects after MES + HS treatment (Figs. 3C and 4C). In MS, mRNA expression for CRP, IL-6, NF-κB and TNF-α showed reductions by 28.0% (p = 0.006), 7.5% (p = 0.36), 22.7% (p = 0.044) and 23.3% (p = 0.030), respectively (Fig. 3D). In T2DM, mRNA expression of CRP, IL-6, NF-κB and TNF-α was decreased by 24.9% (p = 0.046), 15.1% (p = 0.06), 17.7% (p = 0.011) and 33.5% (p = 0.025), respectively (Fig. 4D).

## 4 Discussion

Obesity due to sedentary lifestyle and/or unbalanced diet causes lifestyle-related diseases such as MS or T2DM, characterized by elevated blood glucose, often accompanied by dyslipidemia and hypertension with chronic inflammation resulting from an over-accumulation of visceral fat (Alberti et al., 2005). In this study, we demonstrated that MES + HS, which activates the heat shock response (Morino et al., 2008a, Kondo et al., 2012), significantly improved visceral adiposity, insulin resistance, glucose homeostasis and cytokine levels in males with MS or T2DM.

MES + HS treatment significantly improved insulin resistance in subjects with MS or T2DM. Particularly in T2DM, we have identified significant improvements in HbA1c, FPG, postprandial glucose excursion and insulin resistance. More than half of the T2DM subjects achieved HbA1c less than 7.0%.

Insulin resistance is a central feature of these MS and T2DM, and impaired insulin signaling may be associated with attenuated HSP72 production (Bruce et al., 2003, Kurucz et al., 2002). HSP72 is a cytoplasmic molecular chaperone, which is induced by several stimuli such as heat shock, viral infection or heavy metal contamination etc., and protects cells from apoptosis or cellular dysfunction caused by such cell stresses (Hooper et al., 2014).

HSP72 transcription is regulated by insulin signaling target, glycogen synthase kinase (GSK)-3β.

Phosphorylation of GSK-3β blocks the GSK-3 inactivation of HSF-1 and thereby increases activity of HSF-1 (Hooper et al., 2014).

It is known that HSP72 inhibits JNK activity, resulting in the attenuation of inflammatory signals. However, chronic activation of stress signals down-regulates HSP72 levels through insulin resistance, and results in further activation of inflammatory milieu. This vicious metabolic cycle characterized by systemic inflammation, impaired insulin signaling and attenuated HSR is proposed to occur in MS and T2DM (Hooper, 2009). These components could be improved by the activation of HSR. Indeed, induction of HSP72 by several modalities such as MES + HS, whole-body hyperthermia, muscle-specific HSP72 overexpression, and HSP inducers has been shown to improve insulin signaling in mice, monkeys and humans (Morino et al., 2008a, Adachi et al., 2010, Chung et al., 2008, Kondo et al., 2012, Kavanagh et al., 2011, Literati-Nagy et al., 2009).

The beneficial effects of HSP72 in insulin signaling may be partly explained by the suppression of JNK (Chung et al., 2008). Deletion of JNK1 protects mice from high-fat diet-induced insulin resistance, in part through decreased adiposity. Indeed, suppression of JNK in diabetic mice ameliorates insulin resistance and glucose intolerance (Kaneto et al., 2004). It has also been reported that an HSP72 polymorphism is linked to increased obesity and diabetes risk in humans (Zouari Bouassida et al., 2004). Induction of HSP72 enhances mitochondrial capacity and/or function (Chung et al., 2008, Henstridge et al., 2014), and suppresses JNK activity (Morino et al., 2008a, Kondo et al., 2012). In addition, uncoupling protein-1 mRNA expression in brown adipose tissue was increased in MES + HS-treated diabetic animals (Morino et al., 2008a). Moreover, MES + HS treatment activates AMPK in mice (Kondo et al., 2012). In fact, AMPK α1 knockouts showed increased visceral adiposity with insulin resistance (Zhang et al., 2012). As we have identified the activation of AMPK in monocytes, this may contribute to reduce visceral adiposity. In addition, testosterone treatment ameliorates metabolic abnormalities, including adipocyte hypertrophy, adipose tissue dysfunction, tissue hypoxia and insulin resistance (Maneschi et al., 2012). Heat treatment activates androgen effect especially in lipid peroxidation, indicating that activation of androgen signal through HSR activation may also participate in the beneficial effects of MES + HS. These mechanisms may participate in the reduction of visceral adiposity by MES + HS treatment, which need further investigation.

Increased production of pro-inflammatory cytokines is associated with impaired insulin sensitivity (Tamakoshi et al., 2003). We previously reported that activation of the HSR by MES + HS in diabetic mice was associated with metabolic benefits, accompanied by improvements in inflammatory cytokine productions (Morino et al., 2008a). In this study, we observed similar cytokine changes in human subjects with MS and T2DM. Although this may be partly explained by the reduction in visceral adiposity, we also reported significant reductions in CRP and TNF-α levels by MES + HS in healthy subjects without any body composition changes (Kondo et al., 2010). These results suggest a possible direct association between HSR activation and suppression of systemic inflammation. High serum CRP levels are associated with reduced HSP72 levels (Armutcu et al., 2008), and are positively regulated by NF-κB, which can be attenuated by HSP72. Indeed, we observed a significant increase in HSP72 with reduction of NF-κB nuclear accumulation in monocytes after MES + HS treatment. HSP72 also decreases TNF-α levels by suppression of NF-κB as well. Therefore, induction of HSP72 by MES + HS could decrease inflammatory cytokines through suppression of NF-κB activation. It is also possible that an anti-inflammatory effect of MES + HS could be achieved by AMPK activation, because AMPK α1 deficiency in macrophages markedly increases the pro-inflammatory status (Zhang et al., 2012). As chronic activation of inflammatory status is associated with atherogenesis, anti-inflammatory effect of MES + HS may contribute to limit future vascular complications.

Because visceral fat produces angiotensinogen and increases sympathetic nervous activity, MS and T2DM are often associated with hypertension. In this regard, reduction in blood pressure as a result of MES + HS treatment could be explained, at least in part, by a reduction in visceral adiposity. Recently, it has been shown that heat treatment in high fat-fed mice augmented angiotensin (Shichiri et al., 2000, Alberti et al., 2005, Ohman et al., 2009, Tamakoshi et al., 2003, Morino et al., 2008a, Adachi et al., 2010, Chung et al., 2008), which counteracts against angiotensin II, through Mas receptor/eNOS axis that may improve blood pressure and endothelial dysfunction (Karpe and Tikoo, 2014). Therefore, both reductions in visceral fat and eNOS activation may contribute to ameliorate hypertension upon MES + HS treatment.

Hepatic steatosis is considered to be one of the phenotypes of lifestyle-related diseases. Our results indicate that activation of the HSR by MES + HS improved surrogate markers of fatty liver. Although precise interaction between HSR activation and improvement of fatty liver is still unknown, naringin, a bioflavonoid isolated from grapefruit, activates HSP72 and attenuates hepatic steatosis (Sharma et al., 2011). Alternatively, endoplasmic reticulum (ER) stress attenuation by HSR activation may be involved in this process, because HSP72 enhances ER capacity, and hepatic lipid accumulation is correlated with ER stress (Gupta et al., 2010).

The molecular mechanisms involved in metabolic benefits of MES + HS appear to be similar to those observed in exercise, particularly in HSP72 induction and AMPK activation (Zhang et al., 2009). Indeed, 8 weeks of endurance training in diabetic rats increased HSP72 expression (Atalay et al., 2004). It is also proposed that HSR activators share metabolic pathways associated with exercise with activation of AMPK (Hooper et al., 2014).

Thus, MES + HS treatment may mimic exercise training sharing with similar mechanisms and results.

### 4.1 Limitations

This study was conducted in relatively small number, and the participants were limited to male only in order to preclude menstruation cycle, which may disturb metabolic parameters.

## 5 Conclusion

MES + HS treatment exerts anti-visceral obesity, anti-hypertensive, anti-diabetic and anti-inflammatory effects with no harmfulness, possibly through HSP72 induction and AMPK activation. Moreover, it can also be used in physically handicapped or bed-ridden patients who are unable to follow an exercise regimen. MES + HS could be analogous to exercise training in terms of HSP72 induction and AMPK activation, and might confer additional benefits, such as anti-atherogenic effect. HSR activation by physical MES + HS system may be an alternative and beneficial therapeutic approach to the treatment of lifestyle-related diseases.

## Conflict of interest

The authors report no potential conflicts of interest relevant to this article.

## Author contributions

T.K. designed the study, researched data, contributed to discussion and wrote the manuscript. K.O. and S.Ki. researched data, and contributed to discussion. R.M. and R.G., researched data. M.A.S., S.Ka., M.I., J.K., H.M., and T.M., contributed to the discussion. H.K. reviewed and edited the manuscript. E.A. designed the study, reviewed and edited the manuscript.

## Funding

This work was supported by Japanese government MEXT KAKENHI (Grants-in-Aid for Scientific Research from Ministry of Education, Culture, Sports, Science and Technology, Japan) 2259098701 to TK, 2339024300 to EA. The sponsor of this study has no role in the study design, data collection, data analysis, data interpretation, or writing of the report.

## Figures and Tables

**Table 1:** The subjects’ pre-clinical and biological characteristics.

Background characteristics of the subjects	Metabolic syndrome			Type 2 diabetes mellitus		
Group I	Group II	p value	Group I	Group II	p value
Male/females	20/0	20/0		20/0	20/0	
Age (years)	53.5 ± 1.5	51.3 ± 1.6	0.332	64.6 ± 2.6	71.9 ± 2.6	0.095
Body mass index (kg/m^2^)	26.1 ± 0.5	27.6 ± 0.7	0.093	28.4 ± 0.9	27.4 ± 1.3	0.486
% body fat	26.7 ± 0.9	26.4 ± 0.6	0.890	–	–	–
Waist circumference (cm)	92.8 ± l.l	95.6 ± 1.5	0.120	98.0 ± 2.4	97.7 ± 3.2	0.867
Vsiceral fat area (cm^2^)	147.1 ± 6.8	149.8 ± 10.9	0.774	189.0 ± 11.8	184.4 ± 16.1	0.774
Subcutaneous fat area (cm^2^)	183.5 ± 13.1	200.4 ± 13.1	0.838	152.7 ± 9.2	169.3 ± 20.1	0.521
Systolic blood pressure (mm Hg)	136.8 ± 3.3	133.9 ± 2.6	0.386	138.6 ± 3.4	148.1 ± 5.4	0.205
Diastolic blood pressure (mm Hg)	86.6 ± 2.4	84.8 ± 2.3	0.588	75.2 ± 2.3	74.6 ± 2.8	0.814
Heart rate (beats/min)	69.2 ± 2.1	68.7 ± 1.6	0.862	66.7 ± 2.4	70.4 ± 2.3	0.682
Current smoking (yes/no)	7/13	8/12	N.S.	6/13	4/12	N.S.
Fasting plasma glucose (mg/dL)	107.7 ± 2.7	104.7 ± 2.9	0.457	158.5 ± 9.2	137.7 ± 10.6	0.153
Fasting insulin (mIU/mL)	10.0 ± 0.9	12.9 ± 1.6	0.141	14.5 ± 2.8	14.5 ± 3.3	0.933
HOMA-IR	2.61 ± 0.2	3.36 ± 0.5	0.146	6.03 ± 1.4	5.06 ± 1.3	0.556
QUICKI	0.34 ± 0.01	0.33 ± 0.01	0.195	0.32 ± 0.01	0.33 ± 0.01	0.394
composite WBISI	3.46 ± 0.2	3.04 ± 0.2	0.253	–	–	–
Insulinogenic index	1.02 ± 0.2	1.15 ± 0.2	0.670	–	–	–
HOMA-β	92.9 ± 12.3	1 16.5 ± 14.4	0.231	57.5 ± 11.2	90.6 ± 19.3	0.198
Blood glucose AUC on OGTT (0–2 h) (mg h/dL)	270.3 ± 8.7	274.2 ± 11.6	0.788	–	–	–
Insulin AUC on OGTT (0–2 h) (μIU h/mL)	138.3 ± 14.1	165.4 ± 14.6	0.203	–	–	–
HbAlc (%)	5.69 ± 0.11	5.59 ± 0.14	0.578	7.32 ± 0.17	7.40 ± 0.29	0.861
LDL-cholesterol (mg/dL)	138.0 ± 5.0	124.5 ± 6.9	0.125	121.3 ± 7.1	121.0 ± 8.0	0.814
HDL-cholesterol (mg/dL)	51.6 ± 1.8	48.7 ± 1.8	0.283	50.0 ± 2.6	52.0 ± 4.4	0.811
Triglyceride (mg/dL)	182.1 ± 2.1	163.4 ± 12.1	0.477	145.3 ± 19.8	173.0 ± 11.3	0.228
WBC (/μL)	6009.1 ± 317.9	6026.3 ± 343.7	0.972	54,625 ± 432.3	6643.8 ± 392.0	0.071
RBC (10^4^/μL)	485.7 ± 7.9	502.3 ± 8.0	0.159	482.3 ± 11.8	421.1 ± 14.0	0.334
Hb (g/dL)	15.2 ± 1.2	15.7 ± 0.2	0.116	15.0 ± 0.4	13.9 ± 0.4	0.060
Plt (10^4^/μL)	24.1 ± 0.9	22.6 ± 0.7	0.087	22.4 ± 1.2	21.5 ± 0.6	0.963
BUN (mg/dL)	13.9 ± 0.7	13.0 ± 0.7	0.376	27.5 ± 1.5	26.1 ± 3.1	0.234
Creatinine (mg/dL)	0.78 ± 0.02	0.82 ± 0.03	0.312	1.15 ± 0.12	1.36 ± 0.19	0.130
AST (IU/L)	20.8 ± 0.8	24.9 ± 2.5	0.117	32.2 ± 3.6	24.4 ± 4.3	0.175
ALT (IU/L)	25.3 ± 1.8	35.4 ± 3.7	0.089	35.7 ± 4.5	23.1 ± 4.8	0.085
LDH (IU/L)	l49.0 ± 4.9	163.8 ± 4.5	0.198	228.5 ± 7.7	209.4 ± 9.4	0.113
Adiponectin (μg/mL)	3.15 ± 0.32	2.36 ± 0.34	0.094	3.36 ± 0.93	5.86 ± 1.10	0.345
Leptin (ng/mL)	5.51 ± 0.92	5.77 ± 0.58	0.813	8.38 ± 1.07	9.69 ± 1.31	0.562
Interleukin-6 (pg/mL)	1.48 ± 0.26	1.40 ± 0.1 1	0.761	2.48 ± 0.46	3.50 ± 0.72	0.309
Tumor necrosis factor-α (pg/mL)	1.64 ± 0.21	1.23 ± 0.19	0.278	1.71 ± 0.50	2.32 ± 0.39	0.386
High sensitivity C-reactive protein (ng/mL)	907.14 ± 259.9	607.9 ± 107.1	0.331	1787.7 ± 456.5	2136.9 ± 346.5	0.265
Cystatin C (mg/L)	–	–	–	1.12 ± 0.09	1.65 ± 0.16	0.443
U-albumin creatinine ratio (mg/g·Cre)	–	–	–	329.6 ± 252.5	152.8 ± 64.8	0.307

The subjects' pre-clinical and biological characteristics. Values are expressed as mean ± S.D. or numbers of subjects.

HOMA-IR = the homeostasis model assessment of insulin resistance. QUICKI = quantitative insulin sensitivity check index. WBISI = whole body insulin sensitivity index. HOMA-p = the homeostasis model assessment of p-cell function. LDL = low-density lipoprotein. HDL = high-density lipoprotein. WBC = white blood cells. RBC = red blood cells. Hb = hemoglobin. Plt = platelets. BUN = blood urea nitrogen. AST = aspartic aminotransferase. ALT = alanine aminotransferase. LDH = lactate dehydrogenase.

**Table 2:** Primary outcomes.

Metabolic syndrome							
Adiposity	No treatment				MES + HS		
Baseline	No treatment	Δ no treat	Baseline	MES + HS	ΔMES + HS	p value
Visceral fat area (cm^2^)	148.4 ± 6.4	143.2 ± 6.8	− 5.2	147.4 ± 6.6	134.7 ± 6.4	− 12.7	0.037
SubQ fat area (cm^2^)	191.7 ± 9.5	191.0 ± 9.6	− 0.7	190.3 ± 9.1	187.4 ± 8.5	− 2.9	0.321
Total fat area (cm^2^)	340.1 ± 13.2	334.3 ± 13.0	− 5.9	337.7 ± 12.7	322.1 ± 12.0	− 15.6	0.019
BMI (kg/m^2^)	26.8 ± 0.5	26.7 ± 0.6	− 0.04	26.8 ± 0.4	25.8 ± 0.4	− 0.10	0.328
Wc (cm)	94.1 ± 1.0	93.9 ± 1.0	− 0.2	93.9 ± 1.1	92.9 ± 1.1	− 1.0	0.033

*Glucose control*							
Blood glucose at 0 min (mg/dL)	112.3 ± 1.8	110.1 ± 2.4	− 2.2	112.7 ± 1.8	106.7 ± 2.5	− 6.0	0.029
Insulin at 0 min (μIU/mL)	11.4 ± 1.0	11.7 ± 1.0	0.3	10.4 ± 0.8	9.3 ± 0.7	− l.l	0.049
HOMA-IR	3.2 ± 0.3	3.2 ± 0.3	0.05	2.9 ± 0.2	2.5 ± 0.2	− 0.4	0.024
Insulinogenic index	1.7 ± 0.4	1.4 ± 0.3	− 0.3	1.7 ± 0.4	1.0 ± 0.1	− 0.7	0.042
HOMA-β	87.8 ± 9.0	88.8 ± 7.3	1.0	78.7 ± 6.5	84.0 ± 6.7	5.3	0.732
QUICKI	0.33 ± 0.01	0.33 ± 0.01	− 0.003	0.33 ± 0.01	0.34 ± 0.01	0.01	0.09
Composite WBISI	3.2 ± 0.2	3.5 ± 0.2	0.3	3.3 ± 0.2	4.0 ± 0.3	0.7	0.029
Glucose AUC	334.2 ± 10.6	330.4 ± 12.3	− 3.8	337.2 ± 12.4	325.1 ± 12.9	− 12.1	0.150
IRI AUC	152.6 ± 10.6	148.1 ± 12.2	− 4.5	145.3 ± 9.6	139.4 ± 9.7	− 5.9	0.385
HbAlc (%)	5.24 ± 0.09	5.23 ± 0.08	− 0.01	5.29 ± 0.08	5.22 ± 0.09	− 0.07	0.143

Type 2 diabetes mellitus							

Adiposity	No treatment				MES + HS		

Baseline	No treatment	Δ no treat	Baseline	MES + HS	ΔMES + HS	p value

Visceral fat area (cm^2^)	186.0 ± 9.8	187.1 ± 9.3	l.l	191.8 ± 9.5	179.9 ± 7.0	− 20.9	0.003
SubO fat area (cm^2^)	160.4 ± 11.1	170.0 ± 12.6	9.6	167.8 ± 12.4	169.9 ± 12.5	2.1	0.041
total fat area (cm^2^)	346.4 ± 19.2	357.1 ± 20.7	10.7	359.6 ± 20.6	340.8 ± 17.6	− 18.8	0.005
BMI (kg/m^2^)	27.8 ± 0.8	27.8 ± 0.8	− 0.02	27.8 ± 0.8	27.4 ± 0.8	− 0.4	0.027
Wc (cm)	97.7 ± 2.0	100.5 ± 3.1	2.8	97.9 ± 2.1	95.2 ± 1.8	− 2.7	0.021

*Glucose control*							
Fasting blood glucose (mg/dL)	147.7 ± 7.1	148.2 ± 6.2	0.5	148.6 ± 6.2	133.1 ± 5.4	− 15.5	0.000 l
Fasting insulin (μIU/mL)	14.3 ± 2.1	13.6 ± 1.6	− 0.7	15.0 ± 2.0	11.1 ± 1.6	− 3.9	0.024
HOMA-IR	5.5 ± 0.9	5.1 ± 0.7	− 0.4	5.7 ± 0.9	3.8 ± 0.5	− 1.9	0.009
HOMA-β	73.2 ± 11.4	65.4 ± 8.4	− 7.8	70.8 ± 9.4	66.8 ± 12.4	− 4.0	0.362
HbAlc (%)	7.35 ± 0.17	7.29 ± 0.1	− 0.06	7.27 ± 0.17	6.78 ± 0.14	− 0.49	0.009

The results of MES + HS intervention data compared to no treatment period. Values are expressed as mean ± S.D. Other abbreviations are the same as Table 1.

**Table 3:** Secondary outcomes.

Metabolic syndrome							
Inflammation	No treatment				MES + HS		
	Baseline	No treatment	Δ no treat	Baseline	MES + HS	ΔMES + HS	p value
hs-CRP (ng/mL)	767.9 ± 153.6	1516.8 ± 402.3	748.9	1549.3 ± 420.6	715.0 ± 114.0	− 834.3	0.025
Adiponectin (μg/mL)	2.8 ± 0.2	2.7 ± 0.2	− 0.1	2.9 ± 0.2	3.1 ± 0.2	0.2	0.020
Leptin (ng/mL)	5.6 ± 0.5	5.5 ± 0.6	− 0.1	5.5 ± 0.5	5.0 ± 0.4	− 0.5	0.120
TNF-α (pg/mL)	1.4 ± 0.1	1.3 ± 0.1	− 0.1	1.5 ± 0.1	1.3 ± 0.1	− 0.2	0.020
IL-6 (pg/mL)	1.5 ± 0.1	1.6 ± 0.2	0.1	1.8 ± 0.2	1.3 ± 0.1	− 0.5	0.036

*Blood pressure*							
Systolic blood pressure (mm Hg)	135.5 ± 2.5	134.9 ± 2.5	− 0.6	135.0 ± 2.7	129.3 ± 1.8	− 5.7	0.034
Diastolic blood pressure (mm Hg)	85.8 ± 1.7	86.3 ± 1.4	− 0.5	85.0 ± 1.6	82.6 ± 1.3	− 2.4	0.038
Heart rate (bpm)	68.9 ± 1.4	70.2 ± 1.5	1.3	69.9 ± 1.5	69.6 ± 1.4	− 0.3	0.237

*Others*							
WBC (/μL)	6015.0 ± 239.2	5990.0 ± 287.9	− 25.0	6112.5 ± 254.02	5665.0 ± 256.5	− 447.5	0.037
Hb (g/dL)	15.5 ± 0.2	15.6 ± 0.2	0.1	15.6 ± 0.2	15.4 ± 0.2	− 0.2	0.357
Ht (%)	46.7 ± 0.5	47.7 ± 0.4	1.0	47.2 ± 0.5	47.4 ± 0.4	0.2	0.956
Plt (× 10^4^/μL)	22.8 ± 0.6	22. ± 0.6	0.1	22.4 ± 0.6	23.1 ± 0.7	0.7	0.275
AST (U/L)	22.7 ± 1.3	24.8 ± 1.3	2.1	23.3 ± 1.4	23.1 ± 1.4	− 0.2	0.104
ALT (U/L)	30.5 ± 2.2	31.4 ± 2.1	0.9	30.3 ± 2.2	26.4 ± 1.7	− 3.9	0.012
AST/ALT	0.80 ± 0.04	0.84 ± 0.04	0.04	0.83 ± 0.04	0.94 ± 0.05	0.11	0.023
L/S ratio	0.994 ± 0.001	0.999 ± 0.001	0.005	0.996 ± 0.002	1.092 ± 0.002	0.096	0.003
LDH (U/L)	156.0 ± 3.6	162.4 ± 3.0	6.4	156.4 ± 3.5	161.3 ± 3.5	4.9	0.310
BUN (mg/dL)	13.6 ± 0.5	14.3 ± 0.5	0.7	14.0 ± 0.5	14.2 ± 0.5	0.2	0.139
Cre (mg/dL)	0.80 ± 0.02	0.82 ± 0.02	0.02	0.81 ± 0.02	0.79 ± 0.02	− 0.02	0.010
LDL-C (mg/dL)	131.6 ± 4.4	131.9 ± 4.0	0.3	133.3 ± 4.0	126.1 ± 3.8	− 7.2	0.040
HDL-C (mg/dL)	50.4 ± 1.3	51.4 ± 1.5	1.0	50.4 ± 1.5	50.2 ± 1.5	− 0.2	0.221
TG (mg/dL)	171.5 ± 13.0	164.4 ± 14.6	− 7.1	156.1 ± 10.6	150.0 ± 11.3	− 6.1	0.540

Type 2 diabetes mellitus							

Inflammation	No treatment				MES + HS		

Baseline	No treatment	Δ no treat	Baseline	MES + HS	ΔMES + HS	p value

hs-CRP (ng/mL)	3923.3 ± 1779.0	4206.5 ± 1932.1	283.2	4158.1 ± 1920.7	3969.5 ± 1882.4	− 188.6	0.047
Adiponectin (μg/mL)	4.7 ± 0.6	3.8 ± 0.3	− 0.9	3.9 ± 0.3	4.2 ± 0.5	0.3	0.003
Leptin (ng/mL)	8.9 ± 0.9	10.5 ± 1.1	1.6	10.2 ± 1.1	9.1 ± 0.8	− 1.1	0.003
TNF-α (pg/mL)	2.0 ± 0.3	2.7 ± 0.5	0.7	2.5 ± 0.5	2.0 ± 0.3	− 0.5	0.001
IL-6 (pg/mL)	3.0 ± 0.4	2.8 ± 0.4	− 0.2	2.8 ± 0.4	2.9 ± 0.4	0.1	0.230

*Blood pressure*							
Systolic blood pressure (mm Hg)	143.0 ± 3.3	142.4 ± 3.0	− 0.6	136.7 ± 3.0	129.1 ± 2.5	− 7.6	0.013
Diastolic blood pressure (mm Hg)	74.7 ± 1.8	73.6 ± 1.9	− 1.1	71.7 ± 1.9	66.5 ± 2.0	− 5.2	0.005
Heart rate (bpm)	70.1 ± 1.8	70.6 ± 1.5	0.5	69.9 ± 1.6	68.2 ± 1.8	− 1.7	0.110

*Others*							
WBC (/μL)	6030.3 ± 301.3	5975.8 ± 259.2	− 54.5	6041.1 ± 250.6	6144.1 ± 250.6	103.0	0.512
Hb (g/dL)	14.5 ± 0.3	14.2 ± 0.3	− 0.3	14.3 ± 0.3	14.3 ± 0.2	− 0.04	0.283
Ht (%)	42.0 ± 1.0	41.3 ± 0.9	− 0.7	41.7 ± 1.0	41.3 ± 0.7	− 0.4	0.702
Plt (× 10^4^/μL)	19.3 ± 0.8	18.4 ± 0.7	− 1.1	18.0 ± 0.7	19.8 ± 0.8	1.8	0.175
AST (U/L)	28.5 ± 2.8	27.2 ± 2.2	− 1.3	29.5 ± 2.6	26.2 ± 2.1	− 3.3	0.137
ALT (U/L)	29.7 ± 3.5	29.8 ± 3.0	0.1	29.8 ± 3.0	22.7 ± 1.5	− 7.1	0.068
AST/ALT	1.08 ± 0.06	1.01 ± 0.05	− 0.07	1.09 ± 0.06	1.19 ± 0.07	0.09	0.018
L/S ratio	0.965 ± 0.001	0.973 ± 0.001	0.008	0.973 ± 0.002	1.084 ± 0.002	0.111	0.032
LDH (U/L)	219.5 ± 6.2	204.3 ± 4.3	− 15.2	213.0 ± 5.8	200.5 ± 4.2	− 12.5	0.353
BUN (mg/dL)	21.6 ± 1.8	20.9 ± 1.7	− 0.7	21.0 ± 1.7	19.5 ± 1.6	− 1.5	0.224
Cre (mg/dL)	1.2 ± 0.1	1.2 ± 0.1	0.02	1.2 ± 0.1	1.1 ± 0.1	− 0.1	0.013
LDL-C (mg/dL)	121.2 ± 5.3	121.7 ± 5.0	0.5	121.6 ± 5.1	112.4 ± 4.7	− 9.2	0.007
HDL-C (mg/dL)	51.0 ± 2.5	50.3 ± 2.8	− 0.7	49.9 ± 2.7	52.6 ± 2.6	2.7	0.009
TG (mg/dL)	158.4 ± 12.0	162.4 ± 10.3	4.09 ± 9.07	161.7 ± 11.6	148.0 ± 12.9	− 13.7	0.109
Cystatin C (mg/L)	1.4 ± 0.1	1.4 ± 0.1	− 0.005	1.4 ± 0.1	1.4 ± 0.1	0.007	0.358
U-ACR (mg/g⋅Cre)	199.4 ± 135.8	102.4 ± 54.7	− 37.0	194.4 ± 136.0	89.5 ± 40.7	− 104.9	0.047

The results of MES + HS intervention data compared to no treatment period. Values are expressed as mean ± S.D.

L/S ratio = Liver to spleen ratio on computed tomography.

U-ACR = Urinary albumin creatinine ratio. Other abbreviations are the same as Table 1.

## Appendix A Supplementary data

Supplemental information. Original protocol, final protocol and summary of changes for these trials are indicated.
