# Supplementary material for: Mild Electrical Stimulation with Heat Shock Reduces Visceral Adiposity and Improves Metabolic Abnormalities in Subjects with Metabolic Syndrome or Type 2 Diabetes: Randomized Crossover Trials
Source: eBioMedicine. 2014 Nov 11;1(1):80–9. doi: 10.1016/j.ebiom.2014.11.001 (PMC4457350; doi:10.1016/j.ebiom.2014.11.001)
Supplement: Supplementary file 1 — Supplemental information. Original protocol, final protocol and summary of changes for these trials are indicated. [file mmc1.docx]

Trial Protocols

**Original protocol, final protocol and summary of changes.**

**1) UMIN000001149**

Amelioration of glucose homeostasis and visceral adiposity in metabolic syndrome by mild electrical current and thermo therapy

**1. Original protocol**

Inclusion criteria of Metabolic Syndrome (MS).

Abdominal obesity, given as waist circumference greater than 85 cm with meets 2 criteria in 3 shown below, 
i) Triglycerides: greater than or equal to 150 mg/dL and/or HDL cholesterol: less than 40 mg/dL 
ii) Systolic Blood pressure: greater than or equal to130 mmHg and/or Diastolic blood pressure: greater than or equal to 85 mmHg 
iii) Fasting blood glucose: greater than or equal to 110 mg/dL

Exclusion criteria

i) Past history or current medication of myocardial infarction, angina, apoplexy and arteriosclerosis obliterans. 
ii) Severe wound, infections, pre- or post-operation. 
iii) Severe liver dysfunction (AST and/or ALT: greater than 100U). 
iv) Chronic heart failure, familial dyslipidemia. 
v) Severe diabetic complications. 
vi) HbA1c: greater than or equal to 6.5%. 
vii) Pacemaker carrier.

Intervention 1

12 weeks of MES+HS (4 times a week, 60 min each time), and then 12 weeks of withdrawal, 40 subjects.

Intervention 2

12 weeks of no treatment, and then 12 weeks of MES+HS (4 times a week, 60 min each time), 40 subjects.

Primary outcomes

The amount of visceral adiposity and glucose controls.

Secondary outcomes

Blood pressure, insulin resistance, inflammatory cytokine levels.

Statistical plans

Statistical analysis is performed with SPSS software (IBM, Chicago, IL, USA). All values are expressed as mean ± standard deviation (S.D.). The treatment effects of MET are analyzed by paired *t*-test if data are normally distributed or a Wilcoxon signed-rank test if not. Sequential changes are analyzed by repeated-measures ANOVA. Two-sided *P*-values of less than 0.05 are considered to indicate statistical significance.

**2. 3. Final protocol & summary of changes.**

As we have found that 40 subjects, not 80 can be minimally enough to detect the differences in visceral fat area. Hence, the number of subjects changed from 80 to 40 in this study.

There were no other changes in protocol and statistical plans.

**2) UMIN000003210**

Amelioration of glucose homeostasis and visceral adiposity in type 2 diabetes by mild electrical current and thermo therapy

1. **Original protocol**

Inclusion criteria of Type 2 Diabetes Mellitus (T2DM).

Abdominal obesity, given as waist circumference 
greater than 85 cm

i) Random blood glucose or blood glucose at 120 min on 75g- OGTT: greater than or equal to 200 mg/dL. At least twice.

ii) HbA1c: greater than or equal to 6.5%. At least twice.

iii) Fasting blood glucose: greater than or equal to 126 mg/dL. At least twice.

Exclusion criteria

i) Past history or current medication of myocardial infarction, angina, apoplexy and arteriosclerosis obliterans. 
ii) Severe wound, infections, pre- or post-operation. 
iii) Severe liver dysfunction (AST and/or ALT: greater than 100U). 
iv) Chronic heart failure, familial dyslipidemia. 
v) Severe diabetic complications. 
vi) HbA1c: greater than 10%. 
vii) Pacemaker carrier.

Intervention 1

12 weeks of MES+HS (4 times a week, 60 min each time), and then 12 weeks of withdrawal, 20 subjects.

Intervention 2

12 weeks of no treatment, and then 12 weeks of MES+HS (4 times a week, 60 min each time), 20 subjects.

Primary outcomes

The amount of visceral adiposity and glucose controls.

Secondary outcomes

Blood pressure, insulin resistance, inflammatory cytokine levels and the HbA1c achievement ratio of less than 7.0%.

Statistical plans

Statistical analysis is performed with SPSS software (IBM, Chicago, IL, USA). All values are expressed as mean ± standard deviation (S.D.). The treatment effects of MET are analyzed by paired *t*-test if data are normally distributed or a Wilcoxon signed-rank test if not. Sequential changes are analyzed by repeated-measures ANOVA. Two-sided *P*-values of less than 0.05 are considered to indicate statistical significance.

**2. 3. Final protocol & summary of changes.**

There were no other changes in protocol and statistical plans.

**3) UMIN000007792**

The analysis of monocyte characters on Mild Electrical stimulation with hyperThermia treatment in metabolic syndrome or type 2 diabetes.

**1. Original protocol**

Subjects criteria

10 of MS and 10 of T2DM. Inclusion criteria is the Psame as above.

Intervention

MES+HS treatment (1.4 V/cm, 0.1ms, 55 pps, 42 degrees for 60min), 4 times/week for 4weeks.

Primary outcomes

The expression of CRP, IL-6, NF-kB, TNF-α, HSP72 in monocytes.

Statistical plans

Statistical analysis is performed with SPSS software (IBM, Chicago, IL, USA). All values are expressed as mean ± standard deviation (S.D.). The treatment effects of MET are analyzed by paired *t*-test if data are normally distributed or a Wilcoxon signed-rank test if not. Sequential changes are analyzed by repeated-measures ANOVA. Two-sided *P*-values of less than 0.05 are considered to indicate statistical significance.

**2. 3. Final protocol & summary of changes.**

There were no other changes in protocol and statistical plans.
